# Supplementary material for: Awake fMRI reveals a specialized region in dog temporal cortex for face processing
Source: PeerJ. 2015 Aug 4;3:e1115. doi: 10.7717/peerj.1115 (PMC4540004; doi:10.7717/peerj.1115)
Supplement: Supplemental Information 3 [file peerj-03-1115-s003.pdf]

Consent to Enroll Your Dog in a Research Study

Emory University
Comprehensive Pet Therapy
Dog Star Technologies

<u>Title</u>: **Peering Inside the Canine Mind: Neuroimaging in Awake Domestic Dogs**

<u>Principal Investigator:</u> Gregory S. Berns, MD, PhD, Professor, Dept. of Economics, Emory University
<u>Co-Investigator:</u> Mark Spivak, President, Comprehensive Pet Therapy, Principal, Dog Star Technologies

<u>Introduction</u>
You are being asked to volunteer your dog for a research study. This form is designed to tell you everything you need to think about before you decide to consent (agree) to enroll your dog in the study. **It is entirely your choice. If you decide to allow your dog to participate, you can change your mind later on and withdraw your dog from the study.**

Before making your decision:
- Please carefully read this form or have it read to you
- Please ask questions about anything that is not clear

You can take a copy of this consent form, to keep. Feel free to take your time thinking about whether you would like to have your dog participate.

<u>Study Overview</u>
The purpose of this study is to use functional magnetic resonance imaging (fMRI) to understand how the dog brain responds to different types of stimuli. These include all of the senses (vision, hearing, touch, smell, and taste) as well cognitive functions like memory and emotions. In order to prepare your dog for fMRI scanning, your dog will participate in training sessions along with other dogs at the Comprehensive Pet Therapy (CPT) facilities in Sandy Springs, GA. You will be asked to be present during these training sessions. At the conclusion of these sessions, your dog will undergo fMRI at the Emory University research MRI scanner.

Up to 80 dogs will be recruited for this study. In order to participate, dogs must meet specific height and temperament requirements, which will be assessed at the CPT facilities. Immunization records will be required for all participating dogs.

Metal Implants Certification: If your dog has any metal implanted in its body, it will not be able to participate in this study. Radiofrequency identification chips are okay (such as Home Again). Examples of implants that would exclude your dog from participating include: pacemakers, aneurysm clips, shrapnel, metal fragments, orthopedic pins, screws, or plates, or cochlear implants.

<u>Procedures</u>

Exclusion Criteria: This form will be reviewed with you by a member of the research team. If you agree to enroll your dog, you will be asked to sign this consent form. Your dog will not be able to participate in this study if it has any metal implants, has incomplete immunization records, is currently taking medication, or does not fit the physical and temperament characteristics required to undergo fMRI.

Behavioral Training: To train your dog to be able to stay still in order to undergo fMRI, you and your dog will attend bi-weekly group training sessions at the CPT facilities in Sandy Springs. During these sessions, a professional dog trainer will

help you train your dog to stay still in the fMRI scanner, and to complete simple tasks in the scanner, such as listen to a vocal command associated with a treat. This will also require you to complete exercises at home with your dog between the group training sessions.

Scanning: The ultimate goal of the behavioral training is to train your dog to undergo one or multiple sessions of fMRI, which will measure your dog's brain reaction to different stimuli. Scanning will take place at an Emory University MRI scanner. During the scanning session, your dog will wear ear muffs or ear plugs, and will complete simple tasks that were practiced during behavioral training. On the day of scanning, make sure your dog is using the provided collar, which does not contain any metal. To familiarize your dog with the MRI scanner, you may be asked to visit the facility with your dog prior to the actual scanning.

Recordings: Films, photographs, and audio recordings may be taken throughout the behavioral training and scanning.

Risks and Discomforts

Potential Risks: There is a possibility that your dog could experience damage to hearing due to the noise generated by the MRI scanner. To minimize the chance of this happening, your dog will be outfitted with ear muffs or ear plugs, similar to the procedure used with human participants. There is also the possibility that your dog could escape. If it escaped, it could be injured or killed. It could also cause harm to another person or animal. To minimize this risk, your dog must be leashed during their transportation to the MRI. Your dog must remain leashed until they enter the scanner room, which has three sets of doors between it and the building corridors. As the owner, you will need to be present at all times.

In Case of Injury: An Emory University veterinary technician will be present on the first day of scanning to monitor your dog. However, Emory University does not have veterinary facilities to treat your dog in the event of an emergency. Responsibility for your dog receiving veterinary care will be your responsibility. In the event that your dog is injured or dies during this study, *you will receive no compensation for veterinary care or other costs*.

Benefits

This study is not designed to benefit you or your dog directly. This study is designed to learn more about how the dog brain processes various stimuli. During the course of the study, your dog may learn new behaviors which may or may not benefit you as an owner. The study results may be used to help other owners and their dogs in the future.

Compensation

You will receive payment of $200 for the completion of each successful MRI. A successful scan is determined by the dog not moving such that we acquire enough data for analysis.

Voluntary Participation and Withdrawal from the Study

You have the right to terminate your dog's enrollment in the study at any time. You may refuse your dog's participation in any procedures that you do not feel comfortable with.

The researchers also have the right to stop your dog's participation in this study without your consent if:

- They believe it is in your dog's best interest;
- You were to object to any future changes that may be made in the study plan;
- Your dog is unable to be successfully trained;
- You do not complete necessary training exercises at home.

Contact Information

Contact the research supervisor, Dr. Gregory Berns, at (404) 727-2556:

- if you have any questions about this study or you and your dog's part in it,
- if you have questions, concerns or complaints about the research

You can also contact the Institutional Animal Care and Use Committee Office Director at (404) 727-6212:

- if you have questions, concerns or complaints about the research

Consent

Please, print your name and sign below if you agree to enroll your dog in this study. We will give you a copy of the signed consent, to keep.

I hereby give my consent to all photographs, audio recordings, and/or video recordings taken of me or my dog by CPT, Dog Star Technologies, Emory University staff or their designee. I understand that CPT, Dog Star Technologies, Emory University researchers may take these recordings at any time during the duration of the study. I understand that any such photographs, audio recordings, and/or video recordings become the property of CPT, Dog Star Technologies, or Emory University and may be used for purposes determined by the research team in broadcast and in any electronic media formats.

____

Name of Owner

____ ____________________

Signature of Owner Date Time

____ ____________________

Signature of Person Conducting Informed Consent Discussion Date Time
